# A Robust Kalman Framework with Resampling and Optimal Smoothing

**DOI:** 10.3390/s150304975

**Published:** 2015-02-27

**Authors:** Thomas Kautz, Bjoern M. Eskofier

**Affiliations:** Digital Sports Group, Pattern Recognition Lab, Friedrich-Alexander University Erlangen-Nürnberg (FAU), Haberstr. 2, 91058 Erlangen, Germany; E-Mail: bjoern.eskofier@fau.de

**Keywords:** Kalman filter, fixed-lag smoothing, outlier detection, real-time filtering, non-uniform sampling, parameter estimation

## Abstract

The Kalman filter (KF) is an extremely powerful and versatile tool for signal processing that has been applied extensively in various fields. We introduce a novel Kalman-based analysis procedure that encompasses robustness towards outliers, Kalman smoothing and real-time conversion from non-uniformly sampled inputs to a constant output rate. These features have been mostly treated independently, so that not all of their benefits could be exploited at the same time. Here, we present a coherent analysis procedure that combines the aforementioned features and their benefits. To facilitate utilization of the proposed methodology and to ensure optimal performance, we also introduce a procedure to calculate all necessary parameters. Thereby, we substantially expand the versatility of one of the most widely-used filtering approaches, taking full advantage of its most prevalent extensions. The applicability and superior performance of the proposed methods are demonstrated using simulated and real data. The possible areas of applications for the presented analysis procedure range from movement analysis over medical imaging, brain-computer interfaces to robot navigation or meteorological studies.

## 1. Introduction

The Kalman filter [1] is the optimal filter for linear Gaussian systems in a least mean square sense [2]. However, when the necessary preconditions, *i.e.*, the linearity of the system and Gaussian distribution of the noise terms, are violated, the optimality of the filter output cannot be guaranteed anymore.

**Figure 1.**
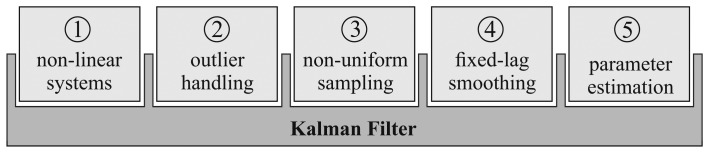
Overview of the presented features and their combination with the original KF.

To overcome these limitations and to enhance the performance of the KF, numerous modifications have been proposed. The adaption of the Kalman framework to non-linear systems [3], Kalman smoothing [4] and the consideration of outliers [5] are amongst the most popular of these alterations. Usually, these modifications are treated separately, and combinations of several methods are disregarded. Additionally, although the KF can be used for non-uniformly sampled data in general, most of the published work describing the aforementioned modifications assume observations that are acquired with a constant sampling rate. Finally, the applicability of the published methods for the estimation of the crucial filter parameters is tightly restricted with regard to the required preconditions. A filter framework that includes the efficient and robust handling of non-linear systems, outliers and non-uniform sampling, while at the same time allowing Kalman smoothing and automated parameter estimation, would make it possible to include all of these modifications and their advantages together. To overcome the irreconcilability of the aforementioned features, we introduce an innovative filtering framework in this paper and a method to estimate the required parameters. An overview of the five presented features and their combination is depicted in Figure 1.

The first and arguably the most prevalent modification of the KF is its adaption to non-linear systems. In contrast to the linear KF, the extended Kalman filter (EKF) [6], the unscented Kalman filter (UKF) [7] and the square-root unscented Kalman filter (SRUKF) [8] are also applicable to non-linear systems. In the EKF, this is achieved via point-wise linearization of the process-model, whereas the UKF and SRUKF both employ the unscented transformation of the state covariance matrix. Non-linearities can also be handled with particle filters, which allow a non-parametric description of probability distributions in the state-space by Monte Carlo approximations [9]. We focus our work on the EKF, due to its high practical relevance.

Secondly, different strategies have been proposed to mitigate the effect of outliers, since they violate the assumption of Gaussian noise. According to Grubbs, “an outlying observation, or outlier, is one that appears to deviate markedly from other members of the sample in which it occurs” [10]. Outliers can occur, for example, in a radar-based localization scenario, where the measurements are disturbed by transient changes from line-of-sight to non-line-of-sight conditions. In an approach presented by Agamennoni *et al.* [11], outliers are taken into account by modeling measurement noise with a Student's t distribution instead of a Gaussian distribution. In the update step of the KF, the measurement noise covariance matrix *R* is replaced by its expected value, which is computed iteratively for each update. This leads to computational overhead, especially when Kalman smoothing needs to be performed, since this iterative algorithm needs to be applied several times for every output value. Moreover, the transfer of this technique to the EKF is not mentioned. A weighted robust KF is presented by Ting *et al.* [12]. Here, a scalar weight is assigned for each data sample, such that a measurement with a lower weight has a smaller contribution for the estimation of the current state. This approach performed as well as a thresholded KF, where measurements are rejected as outliers if their Mahalanobis distance exceeds a certain value. Gandhi and Mili [13] use redundant observations and a threshold on a median-based distance criterion for detection of outliers, at the cost of considerably increased computation time. Other thresholded KFs with the rejection of outliers were reported by Meyr and Spies [14] and by Mirza [15]. However, if information about the statistical properties of the outliers is available, the hard rejection of outliers might not be the optimal solution [16]. A possible alternative is to account for outliers by means of Huberization in a modified correction step. This method is described by Ruckdeschel [17], who presented two robust versions of the KF that are applicable in the presence of endogenous or exogenous outliers. Based on these filters, an outlier-robust Kalman smoother is introduced in [18]. A broader overview about robustness in the state-space context can, for example, be found in the articles by Stockinger and Dutter [19], Ershov and Lipster [20] or Martin and Raftery [21].

Most publications dealing with KFs implicitly or explicitly assume a constant sampling rate for the observations, including multirate systems that comprise several measurement units with a constant sampling rate for each of them [22,23]. Li *et al.* [24] described Kalman filtering for non-uniformly-sampled multirate systems, but outliers and smoothing were not considered. Various possibilities for delayed smoothing of the data have been presented to exploit all available data within a predefined delay, if no real time processing is required. This includes different forms of fixed-point, fixed-interval and fixed-lag smoothing [25]. Fixed-point smoothing can be used when searching for a state estimate at a fixed time, whereas fixed-interval smoothing yields state estimates for a fixed interval of measurements. In many applications (e.g., medical imaging [26], atmospheric process studies [27], target tracking [28]), where the delay that is introduced by smoothing needs to be constant, fixed-lag filtering is the most suitable choice. Considering its wide range of applications, our work focuses on fixed-lag smoothing.

Regardless of the employed modifications of the KF, the estimation of the measurement and process noise covariance matrices is essential for the optimal performance of a KF. This task is considered in various publications. An estimation scheme for a KF based on statistical properties of the innovation sequence is presented by Wang *et al.* [29]. Ghahramani and Hinton [30], as well as Axelsson and Orguner [31] proposed expectation maximization (EM) [32,33] to determine the noise parameters. Furthermore, Bavdekar *et al.* [34] introduced two different approaches (direct optimization and EM) for noise estimation. None of these publications considered the effect of outliers on the parameter estimation. In [11], outliers were taken into account, but here, a constant sampling rate and a linear model were assumed. Moreover, the utilized EM algorithm for parameter estimation was not clarified by the authors.

Despite the vast amount of published work in the field of Kalman filtering, no approach has been published so far for non-linear, outlier-robust Kalman filtering of non-uniformly sampled data with automatic estimation of all necessary filter parameters. Therefore, with the current state-of-the-art, it is not possible to make optimal use of the available data. For example, for non-uniformly-sampled data, one needs to forgo the advantages of smoothing or accept that no fixed delay can be guaranteed.

A filtering framework encompassing the five aforementioned features could improve and facilitate signal filtering in a wide variety of problems, ranging from robot navigation over movement analysis to medical imaging or TV graphics. The purpose of this paper is to introduce an innovative KF framework that combines non-linear systems, outlier handling, non-uniform sampling, fixed-lag smoothing and parameter estimation in a coherent analysis procedure.

First, the basic EKF algorithm will be outlined, and a method for the rejection of outliers will be introduced. Subsequently an EKF-based scheme to create filtered output with a constant rate from non-uniformly-sampled input will be described. Hereafter, these approaches will be combined with fixed-lag smoothing. Finally, a method for the estimation of all filter parameters under the stated conditions will be presented. The efficacy of the developed filter will then be demonstrated and discussed using real and artificial data.

## 2. Methods

### 2.1. The Extended Kalman Filter

The original KF was designed under the assumption of linearity. With the EKF, this approach is transferred to non-linear problems by means of linearization. Like the linear KF, the EKF is a recursive filter comprising a prediction step and a measurement update step.

In the prediction step of each discrete time point *k*, the *a priori* state estimate of point *x̂_k_*_|_*_k_*_−_*_i_* is calculated based on information available up to point *k* − 1. In the update step, the *a posteriori* state estimate *x̂_k_*_|_*_k_* is determined based on information available up to point *k*. The corresponding covariance matrices describing the uncertainty of the state estimates are denoted by *P_k_*_|_*_k_*_−1_ and *P_k_*_|_*_k_*. The function *F*(*x̂_k_*_|_*_k_*, *u_k_*, *T_k_*) characterizes the transition of the state from time step *k* to time step *k* + 1 and specifies the process model. The control input is denoted by *u_k_* and *T_k_* is the possibly non-uniform time between step *k* − 1 and step *k*. The prediction step can be described by:

(1) $${\hat{x}}_{k+1|k}=F\left({\hat{x}}_{k|k},u_{k},T_{k}\right)$$

(2) $$P_{k+1|k}=\Phi_{k}P_{k|k}\Phi_{k}^{T}+{\hat{\Gamma }}_{k}Q{\hat{\Gamma }}_{k}^{T}$$

Here, Φ*_k_* is the Jacobian of the process model for step *k*:

(3) $$\Phi_{k}={\left[\frac{\delta F\left(x,u,T\right)}{\delta x}\right]}_{{\hat{x}}_{k|k},u_{k},T_{k}}$$

The matrix *Q* represents the zero-mean Gaussian process noise. It is transformed using Γ̂*_k_*, which expresses the effect of unknown disturbances on the state dynamics and depends on the choice of disturbance representation [34].

The sensor model, which relates the state vector to a measurement *y_k_*, is represented by the function *h*(*x̂_k_*_|_*_k_*_−1_) with the Jacobian:

(4) $$C_{k}={\left[\frac{\delta h}{\delta x}\right]}_{{\hat{x}}_{k|k-1}}$$

Based on the *a priori* estimate and the measurement at step *k*, the a *posteriori* state estimate is calculated in the update step given by:

(5) $$e_{k}=y_{k}-h\left({\hat{x}}_{k|k-1}\right)$$

(6) $$\Sigma_{k}=C_{k}P_{k|k-1}C_{k}^{T}+R$$

(7) $$K_{k}=P_{k|k-1}C_{k}^{T}\Sigma_{k}^{-1}$$

(8) $$x_{k|k}=x_{k|k-1}+K_{k}e_{k}$$

(9) $$P_{k|k}=\left[I-K_{k}C_{k}\right]P_{k|k-1}$$

The term *e_k_* is called the innovation, and Σ*_k_* denotes the corresponding covariance matrix. *R* represents the measurement noise covariance matrix; *K_k_* is the Kalman gain matrix; and *I* denotes the identity matrix.

### 2.2. Handling Outliers

For Kalman filtering, a Gaussian distribution of the measurement noise is assumed. However, there are cases where this nominal condition is violated by the presence of outliers. It is therefore necessary to make the KF robust against outliers, *i.e.*, it should have good performance under nominal conditions, but also acceptable performance, when these conditions are not met [35]. Outliers corrupt the functionality of the EKF and may even lead to divergence. To avoid such problems, the filter framework must be modified to deal with outliers. We consider the case of Type I outliers [36], *i.e.*, it is assumed that outliers are introduced into the filtering process in the form of outlying observations and are therefore non-propagating. Although there is no general quantitative definition for outliers, a widely-used specification is that from Tukey [37]. Here, extreme outliers are those data points that are not within the interval [*q*_1_ − 1.5 · (*q*_3_ − *q*_1_); *q*_3_ + 1.5 · (*q*_3_ − *q*_1_)], where *q*_1_ and *q*_3_ are the first and third quartiles. For normal distributions, this interval translates to approximately μ± 2.7 σ, where μ and σ are the mean and standard deviation of the underlying distribution.

One possibility to make an EKF more robust is the rejection of outliers based on a threshold-based detection: when an observation is marked as an outlier, it is not included in the filtering process. To detect outliers, we propose to evaluate the Mahalanobis distance (*d_k_*) of the current innovation:

(10) $$d_{k}=e_{k}^{T}\Sigma_{k}^{-1}e_{k}$$

If the Mahalanobis distance of an observation exceeds the predefined threshold α, the current update step is omitted, and the respective measurement is marked as invalid.

In theory, the outlier definition from [37] can be transferred directly into a threshold for the Mahalanobis distance, *i.e.*, α ≜ 2.7. However, in practice, the choice of an appropriate threshold is dependent on the adequacy of the state prediction model and the correct choice of *Q* and *R*, since they influence Σ*_k_*. If the underlying covariance matrix is erroneous, the outliers cannot be detected reliably from the resulting Mahalanobis distance or valid measurements might wrongly be discarded. Therefore, the choice of a pertinent threshold goes along with the estimation of *Q* and *R*, which will be described in Section 2.5.

### 2.3. Conversion of Non-Uniform Sampling

In many applications, an estimate about the state of a system is required at predefined times or with a certain rate. This is the case when observations from different measurement systems with different sampling characteristics need to be synchronized, when transmission standards have to be met or when a constant sampling rate is necessary for further signal processing.

If no observations are available for the predefined instances, a sample rate conversion scheme needs to be employed to guarantee the availability of the necessary information when it is required. To this end, we propose to exploit the prediction abilities of the EKF.

When an observation is usable, the basic recursive steps of prediction and update are applied. State estimates for points in time at which no observation exists are computed from the previous *a posteriori* state estimate, as described in Equations (1) and (2), without a subsequent update step.

We define *M* as the set of time indices *k* for which an observation is available. The set *O* contains all *k* for which a filtered output needs to be computed (*M* and *O* need not be disjoint). The set *M_ν_* contains all *k* for which a valid measurement is available, *i.e.*, where the measurements were not marked as outliers (*M_ν_* ⊆ *M*).

**Figure 2.**
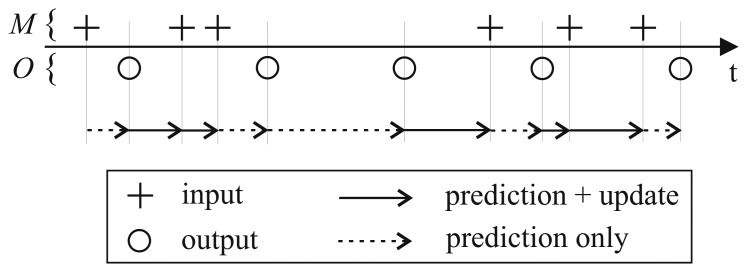
The sample rate conversion scheme uses the prediction abilities of the EKF. At times *t* for which an observation (+) is available, prediction and update are performed. When an output (o) needs to be created without a measurement, only the prediction step is conducted, and the update step is omitted. The set *M* contains the discrete time points for which an observation is available, and the set *O* contains those for which a filtered output is calculated.

The sample rate conversion scheme is summarized in Figure 2. The observation times are assumed to be known, but irregularly spaced. Most commercially available measurement systems provide a timestamp for each observation if the sampling rate is not constant. In the case of uniform sampling, knowledge about the sampling rate is sufficient. The points in time for which an output needs to be created can be chosen either at a constant interval or irregularly spaced. Thus, a predefined output rate can be achieved even when the input is sampled non-uniformly.

The insertion of intermediate prediction steps as described above mitigates a well-known problem: The linearization that is part of the EKF can lead to unstable filter behavior if the time step intervals are not small enough [38]. By adding additional steps, the linearization errors of the EKF can be minimized, since the step width for which the state covariance matrix needs to be propagated is reduced [39].

### 2.4. Fixed-Lag Smoothing for Variable Sampling Rates

For offline data processing, the accuracy of the filtering results can be improved by employing optimal smoothing [25]. An efficient algorithm for optimal smoothing is the Rauch-Tung-Striebel (RTS) smoother [40]. For many applications, offline processing with RTS smoothing is not acceptable, and the filter output needs to be calculated with a constant delay. This can be accomplished using fixed-lag Kalman smoothing. However, to the best knowledge of the authors, no method for fixed-lag smoothing of measurements with variable sampling rates has been reported yet.

For basic RTS smoothing, the smoothed state estimates *x̂_k_*_|_*_N_* and the corresponding covariance matrices *P_k_*_|_*_N_* can be calculated as follows:

(11) $$J_{k}=P_{k|k}\Phi_{k}^{T}P_{k+1|k}^{-1}$$

(12) $${\hat{x}}_{k|N}={\hat{x}}_{k|k}+J_{k}\left({\hat{x}}_{k+1|N}-{\hat{x}}_{k+1|k}\right)$$

(13) $$P_{k|N}=P_{k|k}+J_{k}\left(P_{k+1|N}-P_{k+1|k}\right)J_{k}^{T}$$

where *N* is the index of the point in time corresponding to the last accessible observation and *k* = *N* − 1, …, 0. This algorithm is valid both for uniformly- and non-uniformly-sampled data. It is worth noting that for points in time where no observation is available (*k* ∉ *M_v_*), no measurement update is possible, and *x̂_k_*_|_*_k_* and *P_k_*_|_*_k_* cannot be computed.

For a fixed-lag smoother, Equations (11)–(13) need to be modified, since the data need to be processed with a constant delay *L*, which means that not all *a posteriori* state estimates up to time step *N* are available. Instead, for each *k*, only data up to time step *l_k_* can be used, where *t*(*l_k_*) ≤ *t*(*k*) + *L*. Therefore, for fixed-lag smoothing, we propose to change Equations (12) and (13) to:

(14) $${\hat{x}}_{k|l_{k}}={\hat{x}}_{i|i}+J_{i}\left({\hat{x}}_{i+1|l_{k}}-{\hat{x}}_{i+1|i}\right)$$

(15) $$P_{k|l_{k}}=P_{i|i}+J_{i}\left(P_{i+1|l_{k}}-P_{i+1|i}\right)J_{i}^{T}$$

for *k* = 1, …, *N* and *i* = *l_k_* − 1, …, *k* for each *k*. The initial point is given by *x̂_lk_*_|_*_lk_* and *P_lk_*_|_*_lk_*.

Again, these equations are only valid for points in time where a valid observation is available. If smoothed state estimates need to be computed at points *i* for which no valid measurements are available (e.g., if a constant output rate should be created from a variable input rate and/or if observations were discarded as outliers), the update step is inapplicable, and it follows that:

(16) $${\hat{x}}_{i|i}={\hat{x}}_{i|i-1}$$

and

(17) $$P_{i|i}=P_{i|i-1}\forall_{i}\notin M_{\upsilon }$$

Thus, the *a posteriori* state features *x̂_i_*_|_*_i_* and *P_i_*_|_*_i_* in Equations (14) and (15) can be replaced by the corresponding *a priori* state estimate and covariance *x̂_i_*_|_*_i_*_−1_ and *P_i_*_|_*_i_*_−1_.

To decrease computational overhead, it is sufficient to iterate through *i* ∈ {*l_k_* − 1, …, *k* ∩ (*M_ν_* ∩ *k*)}, because *x̂_i_*_|_*_i_*_−1_ and *x̂_i_*_+1|_*_i_* both incorporate the information from the same observations. Additionally, since an output is not required for all *k*, smoothed estimates only need to be calculated for *k* ∈ *O*.

**Figure 3.**
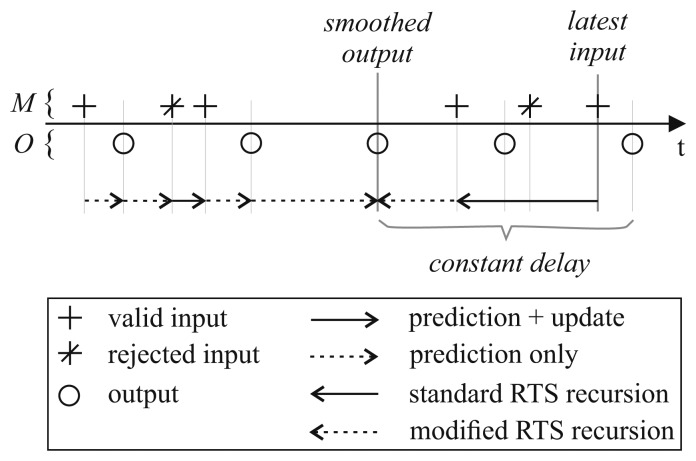
The sample rate conversion as shown in Figure 2 can be combined with outlier rejection and fixed-lag smoothing. Measurements that are marked as outliers are not included in the forward state estimation by skipping the update step. To guarantee a constant delay for fixed-lag smoothing, the recursion is started from the latest valid measurement and is continued up to and including the point in time for which the smoothed output is calculated. The last recursion step is conducted using the modifications described in Equations (16) and (17). Observations that were identified as outliers are ignored in the recursion procedure.

Depending on the available measurements and the defined delay, there may be cases where, for a given output time, there are no valid observations within the delay To ensure continuity in the filtered output, we propose to calculate the corresponding state using the usual EKF equations (including the rejection of outliers) starting from the previous filter output. The filtering scheme described above is visualized in Figure 3.

### 2.5. Parameter Estimation

The performance of an EKF depends crucially on the correct choice of the filter parameters. To avoid inconvenient and possibly imprecise manual tuning of the parameters, an automated estimation approach is indispensable.

We propose a new method, based on the direct optimization strategy presented in [34], to automatically estimate the required filtering parameters, *i.e.*, the process noise covariance matrix *Q*, the measurement noise covariance matrix *R* and the threshold α for the rejection of outliers. Our method does not require reference measurements for parameter estimation, since they are usually difficult to obtain or completely unavailable in practical applications. The problem of finding estimates *Q̂* and *R̂* is solved by formulating it as an optimization problem. The objective function, which needs to be minimized, is the negative of the log-likelihood function of the innovations, which can be formulated as:

(18) $$\left(\hat{Q},\hat{R}\right)=\arg\underset{\left(Q,R\right)}{\min}\left[\sum_{k=1}^{N}\log\left(\text{det}\left(\Sigma_{k}\right)\right)+e_{k}^{T}\Sigma_{k}^{-1}e_{k}\right]$$

This maximum likelihood approach is also applicable for irregularly-sampled systems. It may be noted that the last part of Equation (18) ( $e_{k}^{T}\Sigma_{k}^{-1}e_{k}$) represents the Mahalanobis distance *d_k_* of the innovation. The term $\log(\text{det}\left(\Sigma_{k}\right))+e_{k}^{T}\Sigma_{k}^{-1}e_{k}$ will be summarized as *f_k_*. In the aforementioned publication, it was proposed to solve this problem using standard nonlinear optimization methods, like sequential quadratic programming (SQP). SQP is a gradient-based optimization method and may converge to local minima without reaching the global minimum. Also the EM algorithm for parameter estimation is only guaranteed to converge to a local extremum. To avoid this, we propose to employ a global search method, such as a genetic algorithm (GA) [41], instead. Reasonable bounds for the GA can be identified based on physical considerations of the underlying process and measurement hardware.

To apply the proposed method to the outlier-robust EKF with sample rate conversion, we propose some further alterations: First, since the estimation of *Q* and *R* is based solely on the observations, all steps *k* ∉ *O*\*M* can be omitted. Second, the described approach is only valid for Gaussian noise. Outliers violate the assumption of Gaussianity and, thus, distort the estimation of the true noise parameters by introducing implausibly high values of *d_k_* in the objective function. This can lead to non-optimal estimates of the noise parameters. Additionally, an appropriate threshold needs to be found for the detection and rejection of outliers, which, in turn, depends on the correct estimates of *Q* and *R* (see Section 2.2). This interdependence calls for an approach that allows the simultaneous estimation of the noise parameters and the detection threshold α. Thus, the parameter set that needs to be estimated consists of Θ̂ = (*Q̂*, *R̂*, *α̂*). The necessary modifications of the objective function to estimate not only the noise covariance matrices, but also an estimate for the optimal threshold for the detection of outliers are shown in the following.

The optimal outlier detection threshold allows the rejection of outliers and, at the same time, does not lead to the dismissal of valid measurements. To include this goal in the objective function, we propose to limit the Mahalanobis term in Equation (18) by introducing a penalty term β. If the Mahalanobis distance of the measurement at step *k* is smaller than the detection threshold α, the measurement is not regarded as an outlier, and *f_k_* is calculated as stated before. If the measurement is rejected, because the Mahalanobis distance exceeds the detection threshold, *d_k_* is replaced by β, which is defined as:

(19) $$\beta \left(\Theta \right)=\tilde{d}+1.5\cdot \left(q_{3,d}-q_{1,d}\right)$$

where *d*, *q*_1,_*_d_* and *q*_3,_*_d_* are the median, lower and upper quartiles of the Mahalanobis distances *d*. This means that the penalty term corresponds to the boundary between outliers and inliers in the Mahalanobis distances according to the definition from [37]. As a result, the rejection of valid measurements is penalized, since, in this case, *d_k_* would be smaller than β, *i.e.*, dismissing a valid measurement would lead to an increase of the objective function. The rejection of outliers leads to a decrease in the objective function, because here, *d_k_* would be bigger than β.

The proposed problem formulation can be summarized as:

(20) $$\hat{\Theta }=\arg\underset{\Theta }{\min}\left[\sum_{k\in M}^{N_{M}}\log\left(\text{det}\left(\Sigma_{k}\right)\right)+g_{k}\right]$$

where:

(21) $$g_{k}=\{\begin{array}{ll} e_{k}^{T}\Sigma_{k}^{-1}e_{k} & d_{k}\leq \alpha \\ \beta & d_{k}>\alpha \end{array}$$

and *N_M_* is the number of measurements. The genetic algorithm for the minimization of the objective function is initialized with a constrained random population of possible parameter sets. To evaluate the objective function, the EKF is tested without smoothing with each of these parameter sets. Individuals (parameter sets) that lead to high values in the objective function are discarded. Individuals that lead to low values in the objective function are propagated and modified (using crossover and mutation) by the algorithm to form a new population of parameter sets. This process is repeated, until the improvement in the objective function falls below a threshold. Further details about optimization by means of genetic algorithms can be found in [42].

Assuming the noise characteristics to be constant, parameters can be determined offline using a representative training dataset and then be applied to new data for real-time or fixed-lag filtering. If the system dynamics or the measurement quality vary over time, the proposed method can be used to estimate different parameter sets for different system states and then change between these estimates during runtime. Alternatively, other adaptive methods, as described in [43], can be combined with the proposed filter framework.

## 3. Experiments and Results

### 3.1. Simulated Data

To demonstrate the efficacy of the proposed methods, we analyzed both simulated and measured data. In the case of simulated data, we tested the adequacy of the introduced concept of a penalty term in the objective function for parameter estimation. The calculated parameter estimates were then compared to the true values. Using the determined parameters, the data were filtered with and without fixed-lag smoothing, and the resulting accuracy was investigated. In addition, the performance of the outlier detection method was quantified.

For the simulated data generation, we defined a model that describes the movement of an object in a 2D plane with a constant turning rate and a constant acceleration. As an example, such a model could be used to filter trajectories in sports as they prevail in ice hockey or on a racing or running track.

The state model included the position in two axes (*p_x_*,*p_y_*), the translational speed *ν* and acceleration *a*, the turning rate ω and the orientation ϕ (Euler angle). In our example, only the position in 2D was observable. Since, in the case of trajectory filtering in sports, based on position measurements, no control input *u_k_* is available, it will be ignored in the following.

Two datasets *D*1 and *D*2 with different process noise parameters were created. The process noise was introduced via unmodeled independent changes in *a* and ω (Dataset *D*1) or through *ν*, *a*, ω and ϕ (Dataset *D*2). The state prediction model can be described in compact form as:

(22) $$\left[\begin{array}{l} p_{x,k+1}\\ p_{y,k+1}\\ \upsilon_{k+1}\\ a_{k+1}\\ \phi_{k+1}\\ \omega_{k+1} \end{array}\right]=\left[\begin{array}{c} p_{x,k}+T_{k}\left(\upsilon_{k}+a_{k}T_{k}\right)\cdot \cos\left(\phi_{k}+\omega_{k}T_{k}\right)\\ p_{y,k}+T_{k}\left(\upsilon_{k}+a_{k}T_{k}\right)\cdot \sin\left(\phi_{k}+\omega_{k}T_{k}\right)\\ \upsilon_{k}+a_{k}T_{k}\\ a_{k}\\ \phi_{k}+\omega_{k}T_{k}\\ \omega_{k} \end{array}\right]$$

(23) $$Q=\text{diag}\left[\begin{array}{cccccc} 0 & 0 & \sigma_{\upsilon }^{2} & \sigma_{a}^{2} & \sigma_{\phi }^{2} & \sigma_{\omega }^{2} \end{array}\right]$$

(24) $$R=\text{diag}\left[\begin{array}{cc} \sigma_{x}^{2} & \sigma_{y}^{2} \end{array}\right]$$

The measurement noise was simulated with a standard deviation of $\sigma_{x}^{2}=5\cdot 10^{-3}{\text{m}}^{2}$ in the x-direction and $\sigma_{y}^{2}=5\cdot 10^{-2}{\text{m}}^{2}$in the y-direction. At randomly selected times, outliers were added by changing $\sigma_{x}^{2}$ and $\sigma_{y}^{2}$ to 50 m^2^. The portion of outliers was 5%. To mimic non-uniform sampling, the input time vector was simulated using a Poisson random walk as *T_k_* = 10 ms +1.5 · *τ* · 10^−3^, where *τ* ∼ *Pois*(20).

Each of the simulated datasets consisted of 100 segments of a 30-s length (≈2200 samples each) and was divided into disjoint training (5 segments) and testing subsets (95 segments).

The suitability of the introduction of a penalty term as described in Equation (19) was tested. To achieve the desired effect on the objective function, the penalty term β (Θ) for a given parameter set needs to be bigger than the Mahalanobis distance *d_k_* of valid measurements, thus penalizing the rejection of useful information. In addition, β(Θ) needs to be smaller than the Mahalanobis distance of the outliers to encourage the dismissal of corrupted observations. Therefore, we calculated the percentage of valid measurements for which β(Θ) > *d_k_* and the percentage of outliers for which β(Θ) < *d_k_* as figures of merit (see Table 1). Since the proposed concept also needs to be valid when the correct parameters have not been found yet, we tested all segments of Datasets *D*1 and *D*2 with 1000 randomly chosen combinations of noise parameters. Each single parameter was varied between 10^−2^ times and 10^2^ times its true value, assuming poor *a priori* knowledge of the system.

The noise parameters and the threshold for outlier detection were estimated. This was done using only the training sets. To evaluate the filtering results, only the test sets were used. The parameter estimation and filtering were conducted using the proposed method, *i.e.*, with consideration of outliers during parameter estimation and filtering. Here, the objective function (20) was used. This approach will be referred to as Method *A*. For comparison, parameter estimation and filtering were also conducted without consideration of outliers. Here, the objective function (18) was used. This approach will be referred to as Method *B*.

In both cases, minimization of the objective function by means of SQP yielded no satisfactory results, since in most cases, it did not converge to a global minimum, even when data without outliers were used, and the initial point was chosen close to the true values. Instead, global optimization with GA was employed.

The estimated parameters were compared to the true values. The results for the simulated datasets can be found in Tables 2 and 3. The optimal Mahalanobis distance thresholds for the detection of outliers were estimated to be α_1_ = 41.6 for *D*1 and α_2_ = 10.8 for *D*2.

**Figure 4.**
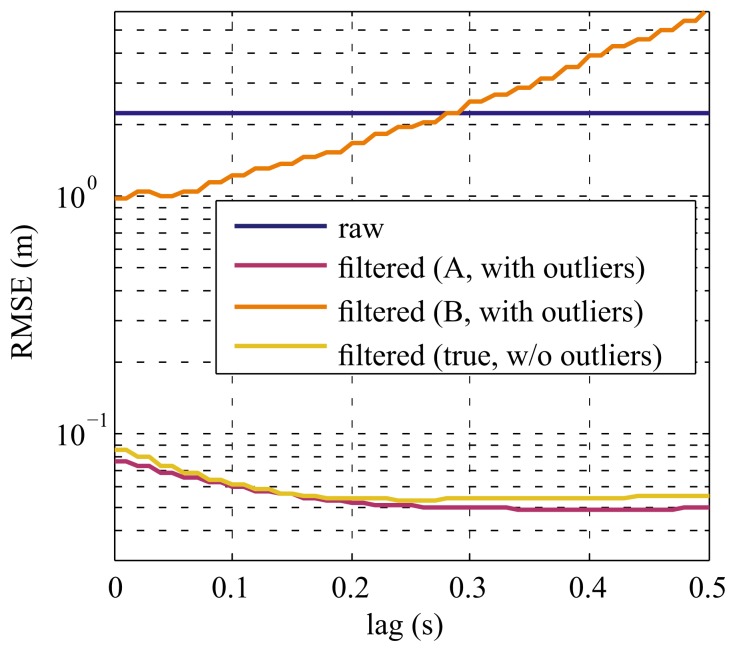
RMSE of Dataset *D*1 (without training set). The blue line shows the RMSE of the unfiltered data (including outliers). The RMSE obtained by filtering the data (including outliers) with the parameters from the proposed method (*A*) is depicted in purple. The orange curve corresponds to the filtering results obtained using the parameter estimation from [34], without special handling of the outliers in the data (*B*). The yellow line represents the results for filtering the data without outliers and with the true simulated noise parameters. The ripple in the filtered curves corresponds to the smallest sampling interval of 10 ms.

**Figure 5.**
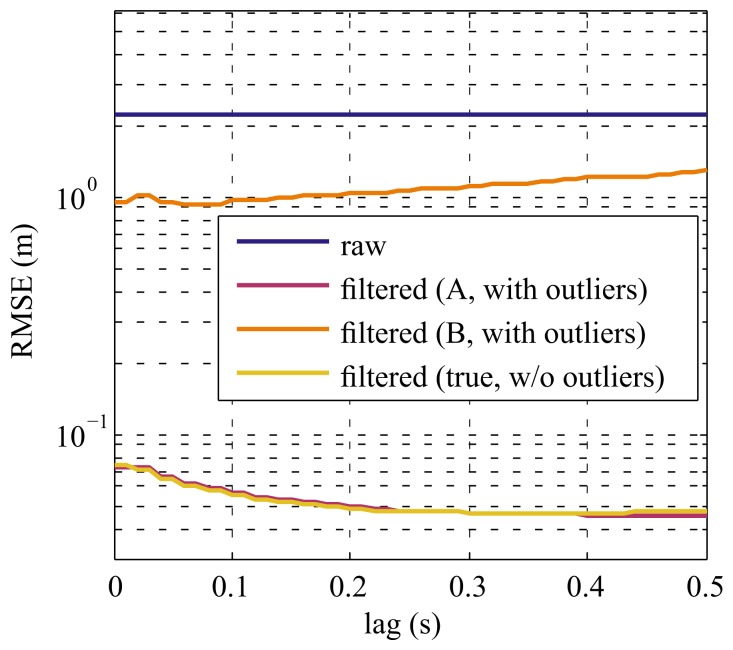
RMSE of Dataset *D*2 (without training set). The results were obtained in analogy to those from Dataset *D*1, depicted in Figure 4.

Using the estimated parameters, the simulated data from the testing sets were filtered with the proposed modified EKF. Moreover, the root-mean-square-error (RMSE) between the simulated ground truth data and the filtered output was calculated for time lags between 0 s and 0.5 s. As a reference, the simulated datasets were also created without outliers and filtered using the true noise parameters. The results are depicted in Figures 4 and 5.

To evaluate the accuracy of the proposed outlier detection method, sensitivity and specificity were calculated for all segments of Datasets *D*1 and *D*2. The results are summarized in Table 4.

### 3.2. Real Data

In addition to the tests with simulated data, the filtering approach was also applied to real data. It consisted of 2D position measurements from a local positioning system (LPS). The LPS employed the frequency-modulated continuous-wave (FMCW) principle. It was comprised of 17 base stations and a reference transponder with fixed and known positions. FMCW signals sent from the mobile target transponder and the reference transponder were used to obtain several time differences of arrival (TDOA) at each sampling time. From these TDOA measurements, the positions of the target were acquired with a non-uniform sampling rate. The time interval between subsequent samples ranged from 5 ms to 400 ms, and the measurements were corrupted by outliers. The LPS was used to observe the movements of a quad bike on a race track. The analyzed LPS data contained approximately 40,000 samples.

To get ground truth measurements for comparison and evaluation, the position of the quad was simultaneously recorded using a Real-Time Kinematic Global Positioning System (RTK-GPS). The measurements from this carrier-phase enhanced differential GPS were evaluated using RTKLIB [44]. The reference antenna for the RTK-GPS was positioned within a distance of less than 1 km from the mobile receiver at all times. Thus, the RMSE of the reference measurements can be assumed to be smaller than 1 cm [45]. The RTK-GPS positions were measured with a constant sampling rate of 5 Hz.

The LPS data were filtered using the presented methods. The output of the filter was synchronized to the reference measurements to allow comparison.

**Figure 6.**
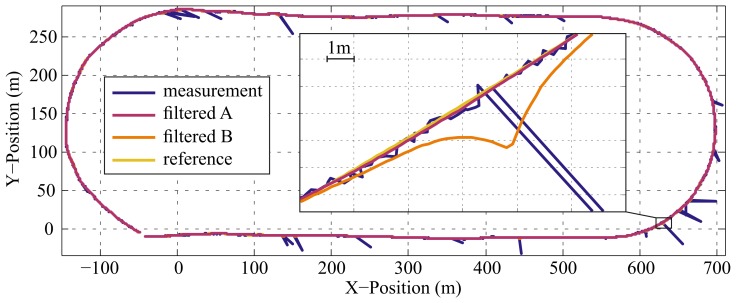
Example plot of the measured and filtered data with magnified detail of the lower right corner of the track. The filtered curves were processed using a constant lag of 500 ms. The measurements were disturbed by Gaussian noise and outliers. Using the proposed method (*A*), the Gaussian noise was reduced, and there was no visible effect of the outliers in the observations. When outliers were not considered in the filtering process (*B*), the filter is susceptible to the outliers, and the filtered curve deviated from the reference. In the depicted case, the deviation added up to several meters.

The estimation of noise parameters and the filtering has been performed both with Method *A* and *B*, with different delays, as described in Section 3.1. A plot of an extract of the measured, filtered (500-ms lag) and ground truth data is depicted in Figure 6.

**Figure 7.**
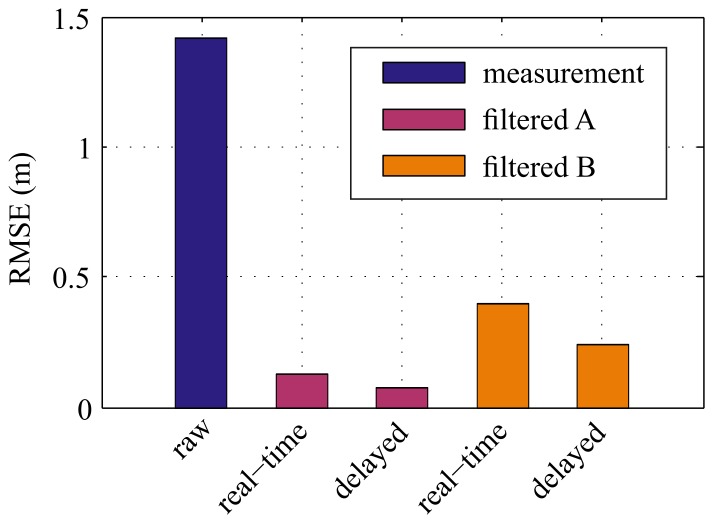
RMSE for filtering real data. The RMSE with respect to the RTK-GPS reference data adds up to 1.42 m for the raw measurements, 0.13 m for the filtered positions (no lag) according to Method *A* and 0.40 m for Method *B*. Using delayed filtering with a constant lag of 500 ms, the RMSE was reduced to 0.07 m for Method A and to 0.24 m for Method *B*.

Since the true noise parameters are not known in this case, they cannot be resorted to for evaluation. Instead, the RMSE serves as the figure of merit. The results in terms of RMSE are visualized in Figure 7.

## 4. Discussion

### 4.1. Simulated Data

The experimental results based on the simulated datasets showed that the proposed concept for the consideration of outliers in the parameter estimation works with sufficient reliability. The introduction of a penalty term mitigates the effect of outliers, even when only poor estimates of the parameters were assumed. The rejection of outliers led to an improvement of the objective function in almost all cases, which compensated for their negative effect. Although there were some cases where the objective function was corrected for only a small fraction of the outliers (worst case: 20.69%), valid parameter estimates could still be found, due to the following two reasons: First, in only 0.24% of the analyzed 200,000 runs, the fraction of outliers that were corrected in the objective function was below 90%. Second, in these cases, the resulting value of the objective function was approximately five-times higher than for the rest, *i.e.*, these parameter sets were far away from the optimal parameters and would be discarded by the optimization algorithm. In the case of valid observations, the objective function was not influenced optimally in around 13% of the runs. This could slow down the convergence of the optimization algorithm.

Using the proposed optimization as expressed in Equation (20), appropriate estimates of the noise parameters in the presence of outliers were determined. The estimated measurement noise parameters acquired with the proposed method (*A*) matched the true values almost exactly, whereas the process noise parameters showed some deviations from the ground truth in both investigated datasets. The noise parameters estimated without special treatment of the outliers (*B*) were considerably overestimated in all cases and differed from the true values by up to two magnitudes. Thus, the negligence of the outliers in the parameter estimation led to an unacceptable overestimation of the real noise parameters.

A drawback of the proposed estimation of the noise parameters by means of a global optimization algorithm, like GA, is the fact that it can become computationally expensive in the case of high order systems. Since convergence to the global optimum is not guaranteed, many function evaluations are usually necessary to find a suitable solution. However, parametrization of the noise covariance matrices as diagonal instead of full matrices can reduce the computational effort in the case of independent noise sources.

Regarding the accuracy of the filtered output, Figures 4 and 5 show the efficacy of the proposed methods: even when non-uniformly-sampled data containing outliers were to be filtered with a constant lag (purple curves), the results in terms of RMSE are as good as if there were no outliers at all in the data (yellow curves). In the case of Dataset *D*1 the RMSE using the proposed filter was actually slightly better than for the reference (which contains no outliers). This is probably caused by the fact that not only the artificially-introduced outliers were rejected, but also some samples that were overly corrupted by the normal measurement noise. This is equivalent to cutting off the tails of the underlying Gaussian distribution and thus, limits the measurement error leading to better filtering results.

The accuracy of the filtered results increased with bigger lags when using the proposed method. This also required increased computation time, since with bigger lags more observations were included in the calculation of each output value.

Finally, the proposed outlier detection method performed robustly. Considering a sensitivity and specificity of more than 94% in all analyzed cases, we conclude that outliers can be found with high accuracy.

### 4.2. Real Data

The results based on real data support the outcomes acquired from the filtering of simulated data. Using the proposed methods, the measured positions were filtered robustly, and clear improvements in terms of RMSE could be achieved. Figure 6 shows that there is no visible effect of outliers on the filtered trajectory for the proposed method (*A*), whereas the filtered curve deviates considerably from the reference measurements when outliers are not considered in parameter estimation and filtering (*B*). Although it is argued in [46] that the hard rejection of outliers is suboptimal, this approach has shown robust operation in the presented simulated and real scenarios, which is in line with other findings in the literature [12,14,15]. The proposed filtering procedure is also compatible with other approaches for the treatment of outliers. Therefore, the results might be further improved, if other robustification methods, (e.g., as described in [17]) are employed in conjunction with the proposed framework.

The use of the presented method for delayed filtering led to further improvements of the filtered signal in terms of RMSE. The fixed-lag filtering allowed enhanced filtering results with a constant time delay and output rate, although the LPS position data were sampled non-uniformly. In contrast with the findings from the simulated datasets, the RMSE decreased even for Method *B* when fixed-lag smoothing was employed. This is presumably due to the smaller amount of outliers in the real measurement data compared to the simulated data; thus, the effect of the outliers was smaller. However, the resulting errors of the filtered output using the presented methods (*A*) was significantly smaller, both with and without smoothing. In this case, the RMSE of the measurements was reduced by 91% without smoothing and 95% with smoothing.

These findings demonstrate that the proposed filtering approach is also applicable under realistic conditions and that considerable enhancements can be achieved.

## 5. Conclusions

In this paper, we introduced a novel Kalman-based filtering framework. It is comprised of several features, namely the handling of non-linear systems, non-uniform sampling and outliers, while at the same time allowing optimal utilization of available information by fixed-lag smoothing and automated parameter estimation. Up to now, only small subsets of these features could be employed at the same time, and the advantages of the others had to be relinquished due to their irreconcilability.

The results based on simulated data exemplify that even in the presence of outliers and with non-uniformly-sampled measurements, suitable noise parameters of linear or non-linear systems can be estimated, and the filtered output can be calculated in real time or with a constant delay. The automated noise estimation makes cumbersome manual parameter tuning redundant. Further, the error of the filtered output is less or equal to the error that would result from filtering data without outliers and with perfectly known system characteristics.

Experimental results based on real measurements testify to the practicability of the newly developed filter in realistic situations and its potential for substantial enhancements of the measured data.

Future work includes investigations on whether and how the parameter estimation can be sped up to increase the viability in case of high-dimensional systems. A robustification of the EM-algorithm for parameter estimation might be a possible solution to reduce the computational cost. Moreover, the transfer of the presented combination of modifications to the UKF and SRUKF will be the subject of future investigations.

With the methods presented in this paper, multiple extensions of the KF become applicable in situations where they could not be used so far, given that the pre-conditions were not met. Thus, the newly presented method expands the boundaries for the application of Kalman-based filtering, which make its advantages accessible even under challenging conditions.

## Figures and Tables

**Table 1.** Relation between penalty term β(Θ) and Mahalanobis distances *d_k_*.

	**β(Θ) > *d****_k_***, *k* ∈ *M****_ν_* (%)		**β(Θ) < *d****_k_*, ***k* ∉ *M****_ν_* (%)	

	**Mean**	**Worst**	**Mean**	**Worst**
*D*1	86.75	78.87	99.21	39.29
*D*2	87.07	77.95	99.59	20.69

**Table 2.** True and estimated noise parameters of Dataset *D*1.

	$\sigma_{x}^{2}$	$\sigma_{y}^{2}$	$\sigma_{a}^{2}$	$\sigma_{\omega }^{2}$
true	5.0 × 10^−3^	1.6 × 10^−2^	8.2 × 10^−2^	5.0 × 10^−3^
*A*	5.6 × 10^−3^	1.8 × 10^−2^	1.7 × 10^−2^	1.4 × 10^−3^
*B*	5.0 × 10^−1^	1.6	7.2	3.9 × 10^−1^

**Table 3.** True and estimated noise parameters of Dataset *D*2.

	$\sigma_{x}^{2}$	$\sigma_{y}^{2}$	$\sigma_{\upsilon }^{2}$	$\sigma_{a}^{2}$	$\sigma_{\phi }^{2}$	$\sigma_{\omega }^{2}$
true	5.0 × 10^−3^	1.6 × 10^−2^	1.5 × 10^−4^	6.4 × 10^−2^	2 × 10^−4^	7.3 × 10^−3^
*A*	5.1 × 10^−3^	1.6 × 10^−2^	9.5 × 10^−4^	3.3 × 10^−2^	3.7 × 10^−3^	5.1 × 10^−3^
*B*	4.9 × 10^−1^	1.6	1.4 × 10^−2^	5.0	1.4 × 10^−2^	6.6 × 10^−2^

**Table 4.** Accuracy of the proposed outlier detection method.

	**Sensitivity (%)**		**Specificity (%)**	

	**Mean**	**Worst**	**Mean**	**Worst**
*D*1	99.86	96.67	99.53	96.62
*D*2	99.27	94.12	99.99	99.86
